# Sustainable behavior in the fishing cards digital game: a comparative analysis across extraction patterns

**DOI:** 10.3389/fpsyg.2025.1507569

**Published:** 2025-04-09

**Authors:** Marlon Alexandre de Oliveira, Giovan Willian Ribeiro, Kalliu Carvalho Couto, Julio C. de Rose

**Affiliations:** ^1^Department of Psychology, Federal University of São Carlos, São Carlos, SP, Brazil; ^2^National Institute for Science and Technology on Behavior, Cognition, and Teaching (INCT-ECCE), São Carlos, SP, Brazil; ^3^Center for Mathematics, Computing and Cognition, Federal University of ABC, São Bernardo do Campo, SP, Brazil; ^4^Department of Behavioral Sciences, Oslo Metropilitan University, Oslo, Norway

**Keywords:** sustainability, digital game, sustainable behavior, age group, extraction patterns

## Abstract

Sustainability is achieved when resources are used to meet current needs without compromising their availability for future generations. This study examined fishing behaviors across age groups using the Fishing Cards digital game. Participants (*N* = 30) played 40 s rounds, using two types of cards to catch fish: one less powerful and the other with greater capacity for resource extraction. The game consisted of two phases. In Phase 1, resources were unlimited, allowing participants to fish freely. In Phase 2, resources were limited and could be depleted, potentially leading to defeat in the game. The research aimed to examine whether InterResponse Time (IRT) for fishing responses changed and how card selection patterns and resource availability influenced participants’ choices. Specifically, the goal was to observe how players achieved sustainability in Fishing Cards when resources became limited. Statistical analysis was conducted using Generalized Linear Mixed Models (GLMM). Findings showed a reduction in the selection of the most powerful card and an increase in IRT during the limited resource phase, indicating more sustainable extraction patterns. As resource availability decreased, participants were less likely to use the most powerful cards. The study contributes to understanding sustainable behavior in experimental analogs and highlights the potential of digital gaming for environmental education and sustainability. Limitations are acknowledged.

## 1 Introduction

The World Commission on Environment and Development (World Commission on Environment and Development (WCED), 1987) defines sustainability as development that meets the needs of the present without compromising the ability of future generations to meet their own needs. Sustainability is a goal which requires policies seeking actions that encourage new ways to use resources without depleting them. Failure to adhere to sustainability practices prioritizes short-term individual gains, often resulting in the accumulation of resources for oneself while harming others who need access to them in the near future.

To prevent unsustainable practices across the natural world and society, global policies, like the UN 2030 sustainable development agenda, commit all member nations to apply 17 Sustainable Development Goals to assure resource preservation for humanity and the planet (SDGs, see United Nations, 2023). The adoption of these objectives aims to reverse the damage caused by human behavior on renewable natural resources’ reserves, such as fishing areas, water reservoirs, and forests. One of these goals, for example, the 14th SDG, “Life Below Water,” is about improving global policies to preserve and maintain our renewable natural resources such as marine biodiversity, rivers, and lakes. These aquatic ecosystems should be used in a balanced manner to allow replenishment over time and avoid environmental actions that can destroy aquatic life and water resources.

The aquatic ecosystems described above are a type of Common-Pool Resource (CPR; see Ostrom, 1990, 2002, 2009; also see de Oliveira et al., 2023; Wilson et al., 2013), which is a resource accessible to all members of a group but is susceptible to overuse and depletion because of its collective sharing. Also, a CPR has two key features: anyone can access the resource without significant barriers and use of the resource by one individual reduces its availability to others. Because of this, extraction of a very large quantity of the resourse can surpass the regenerative capacity, leading to the depletion of resources in the reservoir (see Young and Howatt, 2023), thus characterizing what Garrett Hardin called “Tragedy of the Commons” (Hardin, 1968; Fonseca et al., 2022). Studies have simulated the Tragedy of the Commons in laboratory, often with the use of games. For instance, Camargo and Haydu (2016) simulated fishing areas in a game and found that when there is no feedback about how collective fishing reduces the amount of resources, or when fishing is unlimited, the patterns of resource extraction are maximized. As Hardin said, the principal way to prevent the resource’s collapse is through external intervention (e.g., laws) that restrict impulsive extraction behaviors. When each person acts in self-interest, seeking to maximize their own benefit by using the common resource, an overall decrease in the quality or quantity of the resource may occur, harming everyone in the long run.

Unsustainable use in shared natural renewable CPR contexts have been reverted in laboratory studies that manipulated variables to shift control by short-term individual consequences to long-term positive outcomes for the group. This resulted in an exploitation pattern that did not surpass the renewal capacity of the resource, thus preserving CPR availability (e.g., Brechner, 1977; Camargo and Haydu, 2016). The important methodological features in such studies involved manipulating antecedent and consequent variables to produce changes in the behavior of individuals in order to preserve CPR availability to all users. The experiments of Camargo (2019) and de Oliveira et al. (2023) showed that individuals sharing collective resources with virtual players with whom they could not interact, adopted behavior patterns that led to preservation when short-and long-term consequences were designed to highlight the harmful effects of resource loss (e.g., reducing the frequency of consumption and allowing enough time for resource renewal). Also, in other experiments in which individuals were members of groups whose situations provide context to work together, they managed resources by verbal discussion and or according to rules, as observed in interactions between adults (Nogueira and Vasconcelos, 2015; Vélez and Avalos, 2024) or pairs of children (Koomen and Herrmann, 2018). Previous research tends to focus on the behavior of adults, with the only exception being Koomen and Herrmann (2018), and studies involving children are scarce. Furthermore, the focus on individual choices of children in managing collective resources is underexplored.

Given this gap in the literature, to understand how individual behaviors of children are adjusted to prevent the Tragedy of the Commons in a game context, de Oliveira et al. (2023) employed a video game called Fishing Cards, where players caught fish using cards varying in their extraction capacity. There were two experimental phases: a baseline with unlimited and unshared resources, and an “intervention phase,” where the CPR was limited and shared with virtual players. After successfully completing the intervention phase, participants returned to the baseline condition. The results indicated that baseline extraction was maximized, with very frequent fishing responses using the most powerful card. In the intervention phase, when resources were limited, this pattern led to exhaustion of the CPR. Five out of six children succeeded in moderating extraction patterns, achieving sustainable use of common resources in the digital game. The effect of CPR restriction altered extraction behavior, which shifted to a slower fishing pace and/or prioritization of less powerful cards. Both were strategies used by the players to preserve resources and achieve victory in the game. Upon returning to the condition of unlimited resources, the patterns reverted to those of the first phase, in which resources were unlimited.

Based on de Oliveira et al.’s findings with children, we question whether older participants would similarly moderate their extraction rate and use comparable strategies in the Fishing Cards game. To advance the findings of de Oliveira et al. (2023) to a different population, this study examined fishing-related speed and the density of extraction of a natural renewable common-pool resource using the modified Fishing Cards digital game, designed for an online data collection environment. Teenagers and adults were selected as participants to investigate whether the results from the previous study could be replicated with a different demographic. Specifically, our goals were to: (1) evaluate whether the InterResponse Time (IRT) for fishing responses changed between unlimited and limited resource shared with other players (Independent Variable: resource availability, Dependent Variable: IRT); (2) determine whether participants’ card selection patterns differed (Independent Variable: resource availability, Dependent Variable: card choice); (3) assess whether the amount of available resources influenced their card choices (Independent Variable: resource availability, Dependent Variable: card choice).

## 2 Materials and methods

### 2.1 Participants

Eighteen adults and twelve teenagers (13 females and 17 males) participated in the experiment. They were recruited through online advertising. All participants signed an informed consent form. Adolescents (12 to 17 years old; M = 14.1; SD = 1.9) were recruited after initial contact with their parents and/or guardians and upon obtaining their authorization. Adults (18 to 37 years old; M = 27.5; SD = 4.6) were directly recruited by the experimenter. Before starting the current experiment and after signing the informed consent and/or assent form, participants completed an electronic form regarding their age, date of birth, gender, prior experience with electronic games, the region of the country where they were residing at the time of the research, and their level of education. All the procedures were in accordance with the Human Studies Ethical Review Board of Federal University of São Carlos, Brazil (CAAE: 03865218.3.0000.5504).

### 2.2 Setting

The experiment was conducted remotely using an adapted version of the digital game Fishing Cards (de Oliveira et al., 2023). This version was reprogrammed by the first author to run in web browsers on personal computers (e.g., Google Chrome, Microsoft Edge, etc.) and was accessed through an external link provided by the first author. The first author sent to the participant the link to open on their personal computer. After, the participant was instructed to use the mouse during the game.

### 2.3 Experimental task: fishing card video game

The participants/players were exposed to a video game where they needed to fish with cards representing different fishing technologies for up to 10 rounds, each lasting 40 s. Initially, to win the game, participants needed to fish for 10 rounds in a way that prevented the fish from escaping through whirlpools. The “health points” in the game were indicated by three hearts displayed in the upper right corner of the screen, with one heart being subtracted for each fish that escaped. If the three hearts disappeared, the participant would lose the game and had to start over. The player completed this phase of the experiment by successfully finishing 10 consecutive rounds without being defeated.

To accommodate the remote delivery format of the game and mitigate the potential for player fatigue from prolonged, uninterrupted sessions exceeding 1 h, several modifications were made to reduce the overall difficulty and optimize session length in comparison to de Oliveira et al. (2023). These adjustments were designed to maintain engagement while ensuring that gameplay remained manageable over time. Additionally, the speed of the targets on the game screen was reduced by approximately 30 to 40%, making the game easier to play. Furthermore, this version of Fishing Cards featured a binary selection of cards for capturing fish: the Fishing Rod Card, with lower power, and the Radar Card, offering a more potent option. These changes simplified the game mechanics while retaining the essential elements of challenge and strategy from the previous version. Please, see Supplementary material movies for gameplays details.

#### 2.3.1 Extraction (fishing responses as target behavior)

Through an instant messaging application for smartphones, the first author informed the participant that the game would be entirely automated, meaning that all steps would be performed without the experimenter’s assistance, and instructions on how to play would be presented in the game’s tutorials. Additionally, the experimenter provided instructions on how to log into the system and what the game stages would be (e.g., the player would go through two tutorials, scenarios A and B would have different difficulties), but no information about the game was given. Only a hint was provided: “*Pay close attention to the tutorials and carefully read the game instructions*.” The player was informed that the activity would last about an hour.

Throughout 10 rounds, the player captured fish by selecting cards (Fishing Rod Card/Radar Card) by clicking with the mouse on one of the two panels located at the bottom center of the screen to generate one of these items. This action would collapse the panels, and a card corresponding to the clicked panel would be displayed on the screen. At this point, the player’s task was to click in the card area, hold the left mouse button on this item and move it until hit one of the fish swimming across the screen and then repeat this action. Generating a Fishing Rod Card cost the participant one point, but hitting the target with it earned them +1 point, resulting in a neutral outcome. On the other hand, generating a Radar Card cost five points, but a successful hit returned nine points. All points earned by hitting the targets were accumulated across the rounds, but the Radar Card was the most powerful in the game context, yielding more profit per fish captured than the Fishing Rod Card. If the player clicked on one of the panels more than three times in a row, a cooldown time was triggered by the program, and the player had to wait 8 s to access that type of card again. The purpose of this cooldown time was to prevent an excessive concentration of fishing response rates (e.g., around 20 responses to the same card during 40 s).

The choices of each card used to capture the targets were recorded throughout the experiment, as well as the InterResponse Time (IRT) which indicated the velocity of fishing. The IRT was defined as the elapsed time between two consecutive hits of the card on the fish (see de Oliveira et al., 2023). At the end of each round, the timer was paused and then started again in the next round. Thus, the interval between the last hit in one round and the first hit in the following round also counted as an IRT.

Upon completing the game, the player was instructed to pay attention to a message on the screen indicating: “*Congratulations, you have completed all scenarios and won the game*.” In this event, a button was presented in the upper right corner, and clicking on it rendered a black screen. When this happened, the player was asked to report their success in finishing the procedure to the experimenter.

### 2.4 Experimental design

All participants/players underwent the procedure with the following experimental conditions: Phase 1 and Phase 2. To complete each experimental condition, the player had to complete 10 consecutive undefeated rounds. If a round was lost due to fish escaping or resource depletion, the count would reset. Tutorials were provided between the experimental conditions to teach the necessary elements and performance required to play Fishing Cards. The first author remained in contact with the participants throughout the study to address any major issues related to game crashes, internet disruptions, or check any desire to voluntary drop out by the participant. They were also informed about the possibility of taking a break between rounds if desired. However, no tips or additional instructions were given to any of the participants during the procedure.

### 2.5 Procedure

*Tutorial 1*: After entering a name/nickname and a random code generated by the program, the participant started their gaming session. A screen with four buttons was presented to the player. Only a button with the word “Tutorial” at its center was available; the other buttons, for subsequent conditions were transparent and locked. Clicking on this button displayed sequences of written instructions to progressively teach the required performance during the rounds (refer to Supplementary material A to read the list of instructions). Simultaneously, the player was exposed to situations that required specific actions, such as dragging the Fishing Rod Card to a target to learn how to hit the fish. This taught step-by-step fishing responses in the rounds. Finally, when all tutorial events were completed correctly, the initial screen was presented again, releasing a new button labeled “Scenario A.”

*Phase 1:* Unlimited resources (Scenario A). In this experimental phase, the player had to complete 10 rounds with unlimited resources. To complete it, they needed to capture fish by choosing between two cards representing fishing technologies (fishing rod or radar). After finishing Phase 1, the player returned to the initial screen, and clicking on the “Tutorial 2” button allowed them to proceed with the teaching phase for the new challenges.

*Tutorial 2:* Through visual demonstration and textual instruction, this tutorial taught the player the functionality of a vertical green bar displayed on the left side of the screen. This bar represented the amount of fish in the ocean, and fish capture decreased proportionally the height of the bar; increases in the height also occurred periodically (see Phase 2, below). Subsequently, a new screen introduced the function of new elements: two submarines designated as other players who occasionally captured fish and extracted shared resources with the player (see Supplementary material B for detailed instructions). After completing the tutorial, the player returned to the initial screen with the last button in the bottom right corner accessible, labeled “Scenario B.”

*Phase 2:* Limited and shared common-pool resources (Scenario B). By clicking on the “Scenario B” button, this condition began. As soon as the player entered the Phase 2 screen, they were introduced to the image of two submarines (virtual players) moving on the screen. The virtual players in this phase captured the fish around their area and extracted 10% of the CPR per capture. They were programmed to replicate the fishing response intervals of the participant. In other words, the faster the player extracted CPR, the faster the submarines would do the same. Table 1 presents the IRT range of the virtual players according to the participant’s IRT. When the submarine’s time range was completed, the program waited for 4 s to begin a new update of the participant’s IRT and restate the interval to fish.

**Table 1:** IRT range of virtual players (submarines) as a function of player’s IRT ranges.

Player IRT	Submarines IRT*
≤ 3 s	≥ 2 s to ≤4 s
≥ 4 s to <6 s	≥ 4 s to ≤6 s
≥ 6 s	≥ 6 s to ≤10s

*IRT ranges are in seconds.

Additionally, on the same screen, the participant was introduced to the green bar. It signaled the amount of the natural common-pool access resource (fish) available to the players. The height of the green bar decreased as the participant and the other players captured the fish. However, this bar increased periodically to simulate a renewal of the natural Common-Pool Resource, and a sound was played when this occurred. The total level of the green bar started at 100% CPR, so values of the bar’s level could range from 0 (empty) to 100 (full). Strikes with the cards on the fish were responsible for the resource depletion, as they represented extractions from the CPR. Therefore, every hit with a type of card produced some decrease in the green bar. Specifically, using the Fishing Rod Card reduced this level by 5%, while using the Radar Card decreased it by 15%. To simulate the regeneration of the common-access natural resources the bar would increase by 5% every 2 s. During the interval between rounds, the bar level was paused and could only change when the next round started.

**Figure 1:**
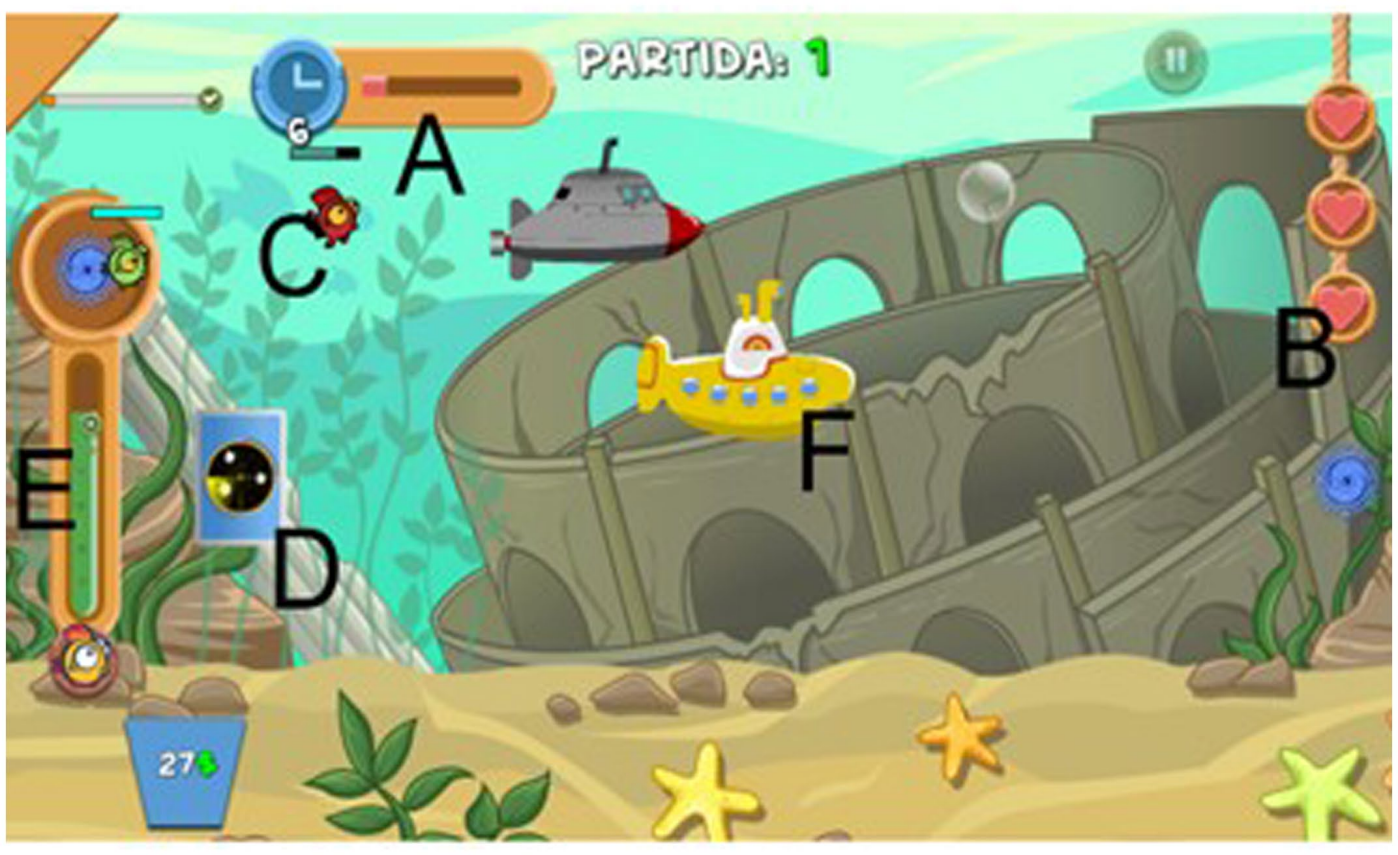
Visual representation of the Fishing Cards game screen during an active round in Phase 2: **(A)** Timer bar indicating the time remaining for completing the round; **(B)** hearts symbolizing the player’s lives; **(C)** fish to be captured, showing the direction in which the targets move and whirlpools (left and right); **(D)** radar card being moved toward the target fish; **(E)** green bar providing visual feedback about level of CPR resources; and **(F)** two virtual players (submarines). “Partida 1” at the top means “Round 1”, i.e., the first round of Phase 2.

If the green bar had a value equal to or less than 30%, semi-transparent red borders were displayed on the screen to signal the risk of CPR depletion. In this situation, if the resource value dropped to 0%, a game loss due to resource depletion was triggered. Regardless of whether the player lost by letting the fish escape from the screen or by depleting all of the CPR, they repeated Tutorial 2 and returned to Phase 2. When the player successfully completed 10 consecutive rounds, the experiment was terminated, and a black screen was presented. Figure 1 shows the green bar, the submarines, and all other elements presented when playing (i.e., Cards, Fish, Live Hearts and Timer; to more details see de Oliveira et al., 2023).

We implemented a within-subject design, allowing for a direct comparison of participant’s performance between Phase 1 and Phase 2 (Cooper et al., 2007; Kazdin, 2011). Following an initial phase of unlimited resources, we introduced the independent variable: the availability of CPR (Common-pool resources) in the form of limited renewable natural resources within the virtual environment of the Fishing Cards digital game. This was indicated by the green bar and the presence of two additional players (submarines). To measure extraction during fishing responses, we established two dependent variables: the fishing velocity, defined as the IRT, and the card choices (Fishing Rod or Radar Card).

### 2.6 Data analysis

Participants were divided into two age groups: teenagers (*N* = 12) and adults (*N* = 18). The data were analyzed to address three main objectives:

Objective 1: Evaluate whether the InterResponse Time (IRT) for fishing responses changed when transitioning from an unlimited resource condition (Phase 1) to a limited, renewable shared resource condition (Phase 2).

Objective 2: Determine whether participants’ card selection patterns differed between Phase 1 and Phase 2.

Objective 3: Assess whether the amount of resources available to participants (as indicated by the resource green bar) during Phase 2 influenced their card choices.

Additionally, for all objectives, we examined whether participants’ age group (teenagers vs. adults) significantly affected the outcomes.

This experiment employed a within-subject design, exposing participants to two conditions (Phase 1 and Phase 2), and a between-subject design, categorizing them into adult and teenager groups. The analysis used the data from the 10 rounds of Phase 1 e the last 10 rounds of Phase 2, where participants achieved 10 consecutive victories.

To assess the impact of the experimental phase, participants’ age, and resource levels (independent variables) on the cards used and participants’ IRT (dependent variables), different Generalized Linear Mixed Models (GLMM) were employed. Specifically, we tested the influence of Phase 2 exposure on participants’ IRTs (Objective 1), the effect of this phase on the proportion of Radar card usage (Objective 2), and whether the level of resources displayed during Phase 2 influenced participants’ card choice (Objective 3). We also explored potential age-related differences. All models incorporated the participant ID and the response index (1st, 2nd, 3rd, etc.) as random factors. The null hypotheses were rejected when *p*-values were less than 0.05. The analyzes were conducted on the R Studio software (Version 2023.9.1.494; Posit team, 2023) using the packages tidyverse (Wickham et al., 2019), lme4 (Bates et al., 2015), emmeans (Lenth, 2021), and performance (Lüdecke et al., 2021). Scripts and data are available in the Supplementary material. For each objective, different model configurations were tested and compared to identify the best fit. Table 2 presents the specifications for each model. Comparisons included models with Phase and Age as main effects with and without interactions, as well as models with Phase as the sole main effect. For Objective 1, given that IRT is a non-negative continuous variable, the Gamma distribution was used, and models with “inverse” and “identity” link functions were compared. For Objectives 2 and 3, where card choice was a nominal binary variable, the Binomial distribution was applied, comparing models with “logit” and “probit” link functions. The model with the lowest Akaike Information Criterion (AIC) value was selected as the most appropriate for each objective.

**Table 2:** Models evaluated for each objective, detailing the main effects, interactions, the distribution family applied, the link function used, and the Akaike Information Criterion (AIC) value.

Objective	Main effects	Interaction	Distribution family	Link function	AIC
1	Phase and Age	Phase x Age	Gamma	Inverse	44704.0
	Phase and Age	–	Gamma	Inverse	44702.3
	**Phase**	–	**Gamma**	**Inverse**	**44700.6**
	Phase and Age	Phase x Age	Gamma	Identity	-
	Phase and Age	–	Gamma	Identity	-
	Phase	–	Gamma	Identity	-
2	**Phase and Age**	**Phase x Age**	**Binomial**	**Logit**	**5530.2**
	Phase and Age	–	Binomial	Logit	5538.5
	Phase	–	Binomial	Logit	5537.2
	Phase and Age	Phase x Age	Binomial	Probit	5531.2
	Phase and Age	–	Binomial	Probit	5540.7
	Phase	–	Binomial	Probit	5539.3
3	Resource and Age	Resource x Age	Binomial	Logit	1546.0
	Resource and Age	–	Binomial	Logit	1544.3
	**Resource**	–	**Binomial**	**Logit**	**1543.6**
	Resource and Age	Resource x Age	Binomial	Probit	1548.7
	Resource and Age	–	Binomial	Probit	1547.4
	Resource	–	Binomial	Probit	1546.7

The model with the lowest AIC for each objective was selected as the best fit and is marked in bold. Models in Objective 1 with an “identity” link function failed to converge and therefore did not calculate the AIC.

For the analysis of IRT (Objective 1), the best model included Phase as the sole main effect and used an “inverse” link function. It is important to highlight that in the other models tested for this objective, which included Age as a factor, Age was not significant either as a main effect or in interaction with Phase. When exploring card usage patterns (Objective 2), the optimal model incorporated Phase and Age as main effects, along with their interaction, and applied a “logit” link function. For the influence of resource levels on card choice (Objective 3), the selected model included only Phase as a main effect, also with a “logit” link function. Similarly to Objective 1, in the remaining models tested for Objective 3, Age was not significant as a main effect or in interaction with Phase.

To ensure the reliability of the selected models, we conducted additional diagnostics. The normality of the random effects (participant ID and response index) was evaluated through Q-Q plots, which confirmed that the random effects followed a normal distribution. These plots are provided in the Supplementary material file “qq_plots_random_effects.pdf,” with the corresponding code included in the “script.R” file. We also checked the models for potential dispersion issues. No signs of overdispersion were identified, and the methods used for this evaluation are detailed in the same script file.

The normality of the random effects (participant id and response index) was checked for all the selected models. Inspection of Q-Q plots showed that random effects were normal for all models. The Q-Q plots were included in the Supplementary material file “qq_plots_random_effects.pdf” and the code to generate then is included in the “script.R” file. The selected models were also checked for overdispersion, with none of them presenting overdispersion. The “script.R” file contains the code used to assess overdispersion.

## 3 Results

The results are summarized in Table 3, which details the selected models for each objective. To facilitate interpretation, the outcomes for each objective will be addressed in separate sections below.

**Table 3:** Summary of the selected models for each objective, including the model effects, estimates, standard errors, test statistics, *p*-values, and the residual degrees of freedom.

Objective	Effect		Estimate	Std. error	Test statistic	*p*	df. resid
1	Main	Phase	−0.0024	0.0005	*t* = −4.59	**<0.001**	5,059
2	Main	Phase	−1.1713	0.0861	*z* = −13.6	**<0.001**	5,058
		Age	0.2894	0.5786	*z* = 0.5	0.617	
	Interaction	Phase x Age	0.4764	0.1476	*z* = 3.23	**0.001**	
3	Main	Phase	0.0186	0.0041	*z* = 4.53	**<0.001**	1977

Statistically significant effects (*p* < 0.05) are highlighted in bold.

### 3.1 Objective 1

**Figure 2:**
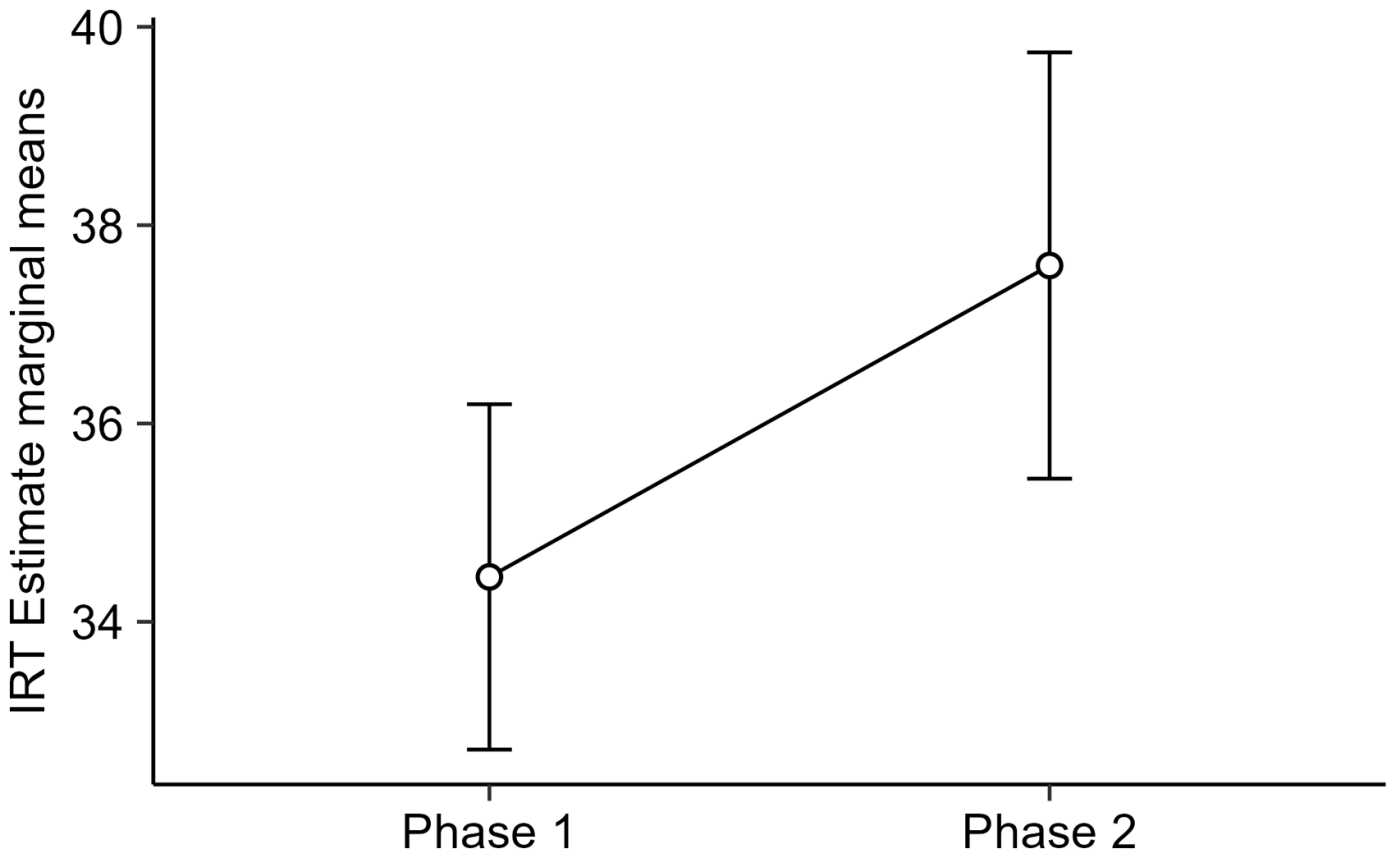
Estimated marginal means of participants’ IRT in each phase. Results were back-transformed from the inverse scale. Error bars represent a 95% confidence interval for the mean.

A GLMM tested whether participants’ IRT differed according to the experimental phases (within factor) and the age of the participant (between factor). To address the distribution skewness typical of timing data, we used the gamma distribution with an inverse link function. The model showed a significant main effect for phase, with IRTs increasing from Phase 1 to Phase 2. Figure 2 represents the model estimates.

### 3.2 Objective 2

**Figure 3:**
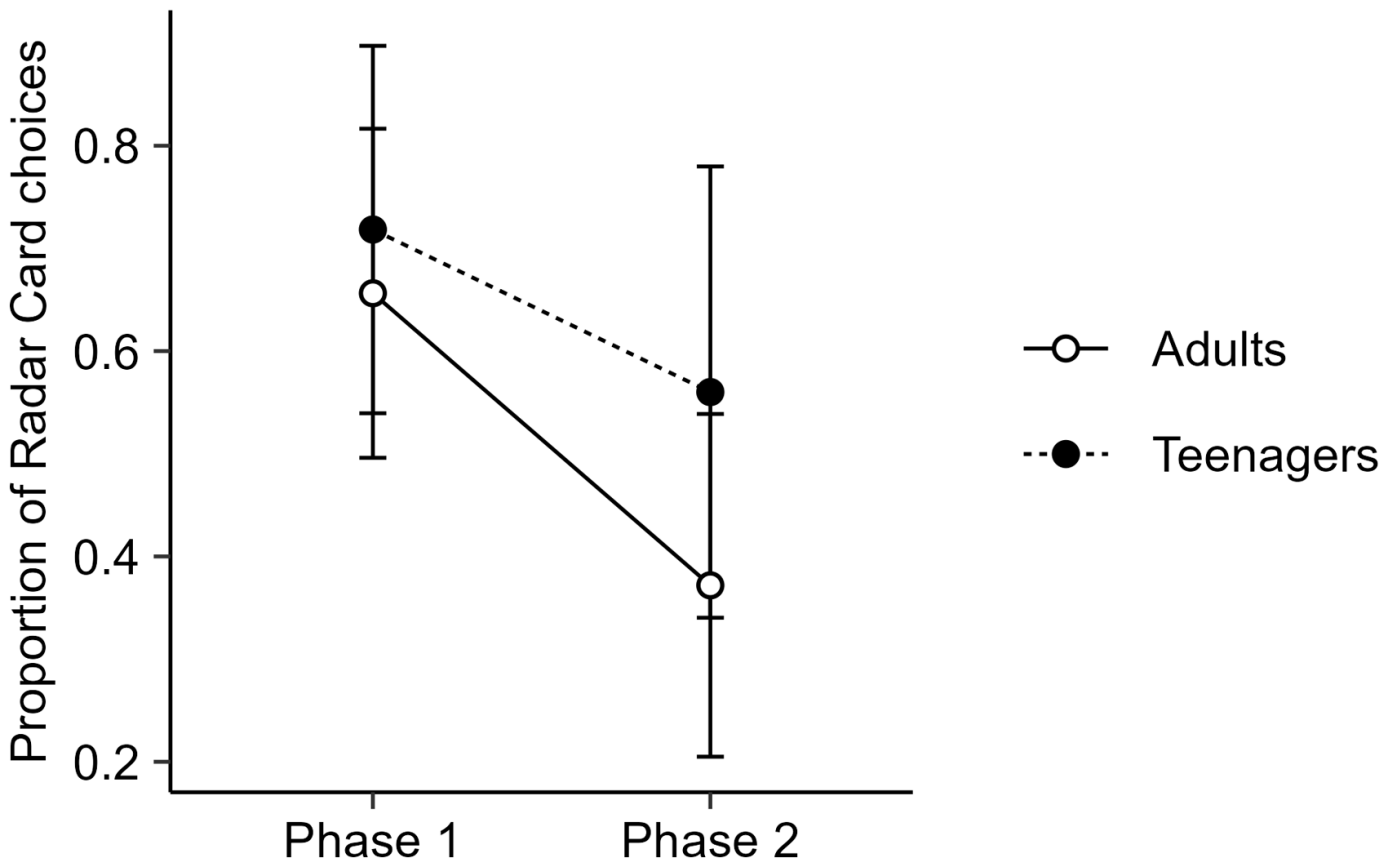
Estimated proportion of Radar Card choices in each phase and according to participants’ age. Results were transformed from a log (odds ratio) scale to a proportion scale. Error bars represent a 95% confidence interval for the mean.

A logit distribution was employed in a GLMM (i.e., a logistic regression model) to assess the effect of phase and age over participants’ card choice. The model showed that the proportion of Radar Card choices decreased from Phase 1 to Phase 2, and an interaction effect indicates that the decrease was more pronounced for adults. There was no significant effect for age alone. See Figure 3 for model estimates.

### 3.3 Objective 3

**Figure 4:**
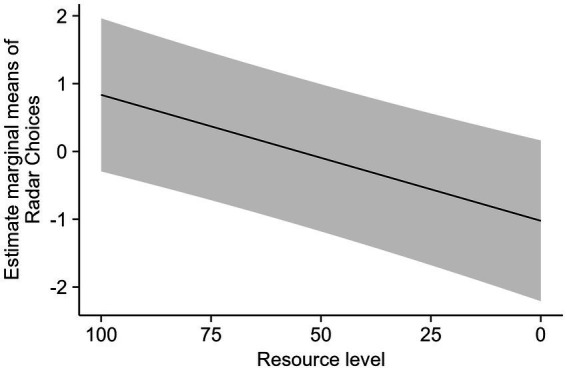
Estimated marginal means of Radar Card choices according to resource levels during the last 10 rounds in Phase 2. Results are given on the logit (log[odds ratio]) scale. The shaded area represents a 95% confidence interval for the regression line.

A logistic regression GLMM tested whether the resource level could predict participants’ card choices. Since the resources bar was available to the participant only during Phase 2, data from Phase 1 were excluded from this analysis. It was found that the resource level significantly predicted the use of the Radar Card. Figure 4 shows the estimates of the model.

## 4 Discussion

The results of the present experiment responded to the objectives: in Phase 2, when a natural renewable resource was limited and shared, there were changes in fishing response velocity (indicated by the IRT) and card choices in adults and adolescents. Data showed a decrease in choices of the Radar Card and an increase in IRT when the participants achieved victory. As far as we know, this is the first study to achieve similar sustainable behaviors to avoid depletion of CPR across different age ranges, involving teenagers and young adults, showing more sustainable patterns, similar to those described by previous studies that have investigated experimental scenarios analogous to the tragedy of the commons (e.g., Brechner, 1977; Camargo and Haydu, 2016; Camargo, 2019) in university populations as well as in children (Koomen and Herrmann, 2018; de Oliveira et al., 2023). The clear differences in extraction patterns between phases confirm the effects of conditions established when limited and shared collective resources were presented.

Camargo (2019) manipulated the points lost based on whether the IRT was below or above the optimal level, while de Oliveira et al. (2023) adjusted the reinforcement ratio between low-and high-density extraction cards. Similar to the results of the previous experiments by Camargo and de Oliveira et al., the fishing velocity of participants in this study was controlled by elements signaling limited shared resources. As a result, participants’ fishing velocity tended to decrease in response to antecedent stimuli (such as the falling green bar during rounds) and negative consequences, such as resource decrease through excessive extractions or game termination due to resource depletion. The increase in mean IRTs for teenagers and adults, as depicted in Figure 2, highlights this effect, answering the question set by Objective 1.

Results about card choice are consistent with de Oliveira et al. (2023) intervention phase, similar to Phase 2 in the present experiment. In the present study we observed a decrease in the proportion of Radar Card choices for both adults and teenagers (see Figure 3). With a more prominent decrease in adults, the scenario of limited CPR changed the behavior to use the card with less extraction capacity, showing a strategy to lower resource extraction, which was a sustainable behavior that could balance the opposing demands of the game: capture fish to avoid game loss by fish escape, whereas moderating the amount of capture to avoid loss by depletion of fish.

Regarding Objective 2, in general, teenagers and adults had decreased probabilities of choosing the Radar Card as the resources diminished. This result can be interpreted as a control of the behavior by a stimulus that signal resource depletion (green bar). This is similar to findings of studies of resource management (e.g., Brechner, 1977; Camargo and Haydu, 2016; Young and Howatt, 2023). The tendency to maximize short-term gains by excessive extraction may be attenuated when the game provides feedback about decrease in resources.

Addressing Objective 3, the tendency to prioritize immediate gains and short-term time use in Phase 1 by selecting Radar Cards reflects the impulsivity described by Rachlin (1995, 2000). Rachlin defines impulsivity as the preference for immediate rewards over delayed, larger benefits, often resulting in suboptimal long-term decision-making. Interestingly, adolescents and adults exhibited similar behaviors when using Radar Cards, suggesting a shared tendency to prioritize immediate outcomes, similar to the tendency for accumulative gains described by Borba et al. (2014), Platt (1973), and Young et al. (2013). To examine this further, we introduced Scenario B, where resources are finite, and impulsive selection of Radar Cards leads to game over. This scenario demonstrates how feedback about resource levels, shared with other players, alters the immediate rewards of Radar Card selection by changing the consequences of available choices. These extraction patterns help maintain resource levels with less impact, create opportunities for sustainable resource renewal, and mirror real-life challenges in managing common-pool resources (CPRs), such as fishing areas, where impulsive decisions can deplete resources over time.

Furthermore, the sustainable extraction patterns were confined to Phase 2, specifically targeting fishing responses to preserve resources across all rounds within the virtual CPR context. Despite recognizing the importance of demonstrating moment-to-moment behavioral changes to counter the grim predictions outlined in the Tragedy of the Commons for finite resources, our study did not explore the impact of the learning process on participants’ environmental attitudes after exposure to new experimental scenarios. Therefore, participants’ self-reports could provide insight into how the game influenced their views on CPR preservation during the experimental situations. This raises questions about whether certain elements should be incorporated to create opportunities for sustainable reflection based on previous gameplay experiences.

As all experiments have methodological limitations, this study has its own. We recommend future studies with a larger participant sample to investigate the effects of age, including a broader range of players of different ages. From a perspective aimed at evaluating cooperative behaviors, further research could be conducted in a lab setting and allow communication between players, thereby enhancing the ecological validity of sustainable strategies through verbal interaction. The literature suggests that dialog enables groups to self-manage (Dietz et al., 2003; Ostrom, 2009; Wilson et al., 2013; Nogueira and Vasconcelos, 2015). The present experiment, as well as de Oliveira et al. (2023) showed that a sustainable pattern of extraction may be achieved without dialog; nevertheless, it will be interesting to study its effects in the context of this game. Other parameters could also be adjusted to verify if changes in one variable are key features in resource management levels. For example, game elements such as fish presentation on the screen, rates of resource bar increase or decrease, and scoring could be adjusted differently to determine their impact on CPR use and ensure improvements in accurate measurements.

The extraction patterns observed in the present study indicate that digital games designed to simulate the consequences of resource uses over short time periods should align with actions to promote sustainable development (see Brundtland, 1987). As de Oliveira et al. (2023) suggested, the Fishing Cards video game could be a promising educational tool, as it allows children to experience, in a playful context, the consequences of irresponsible use of common-pool resources, through immersion in the game. Similar to findings of de Oliveira et al. with children, Phase 2 of the present experiment replicated sustainable extraction patterns exhibited by both teenagers and adults. Thus, Fishing Cards might also serve as an effective intervention tool with more mature populations.

## Data Availability

The datasets presented in this study can be found in online repositories. The names of the repository/repositories and accession number(s) can be found in the article/Supplementary material.
