# Supplementary material for: Sustainable behavior in the fishing cards digital game: a comparative analysis across extraction patterns
Source: Front Psychol. 2025 Apr 9;16:1507569. doi: 10.3389/fpsyg.2025.1507569 (PMC12015162; doi:10.3389/fpsyg.2025.1507569)
Supplement: Supplementary file 1 [file Data_Sheet_1.zip › Supplementary material presentation Data Sheet/qq_plots_random_effects.pdf]

Objective 1

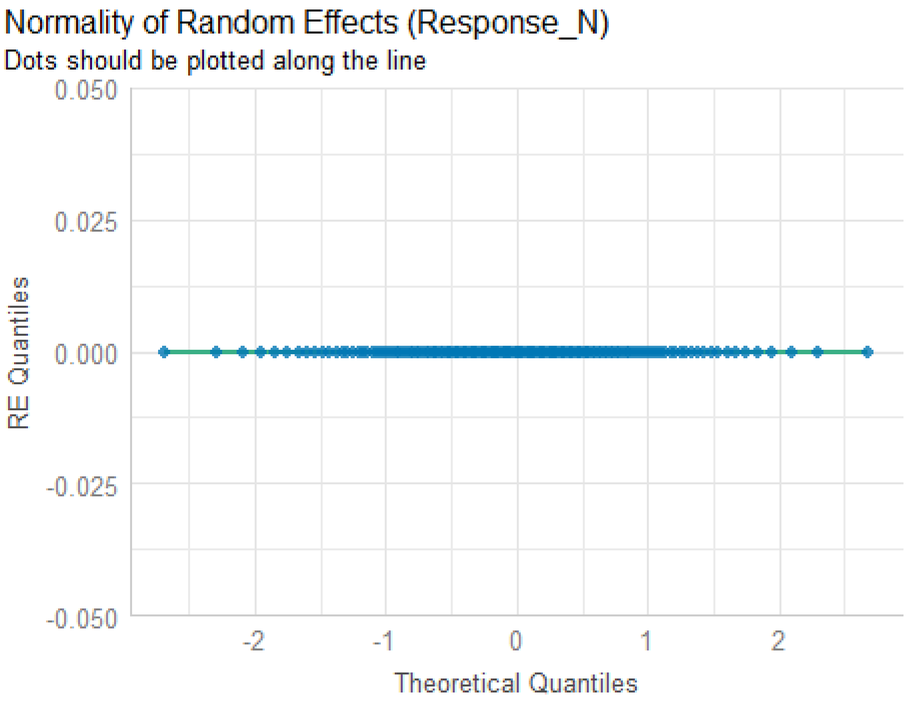

Normality of Random Effects (Response_N)
Dots should be plotted along the line
0.050
0.025
0.000
-0.025
-0.050
RE Quantiles
-2
-1
0
1
2
Theoretical Quantiles

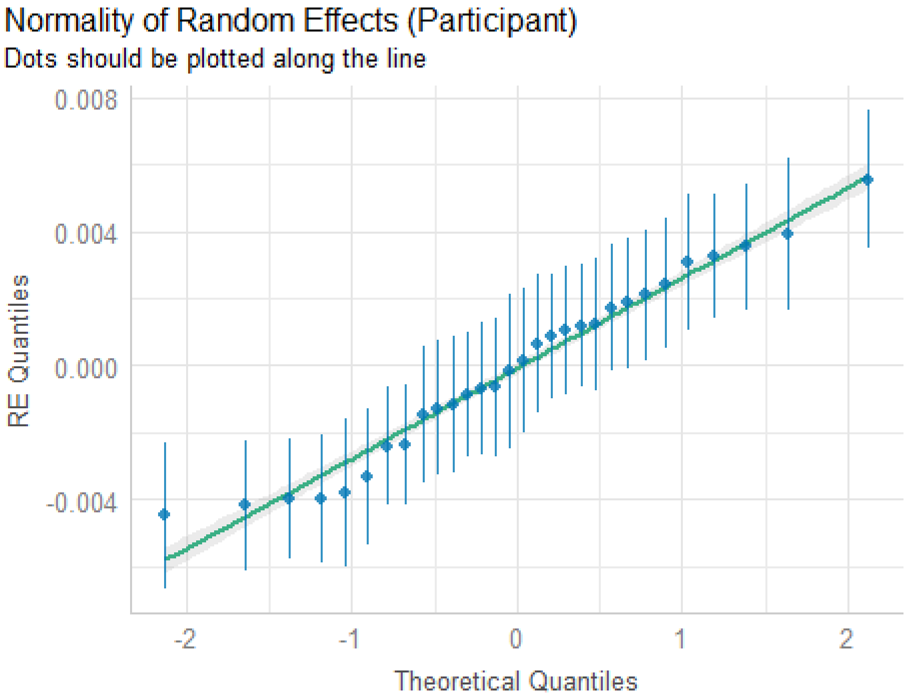

Normality of Random Effects (Participant)
Dots should be plotted along the line
0.008
0.004
0.000
-0.004
RE Quantiles
-2
-1
0
1
2
Theoretical Quantiles

Objective 2

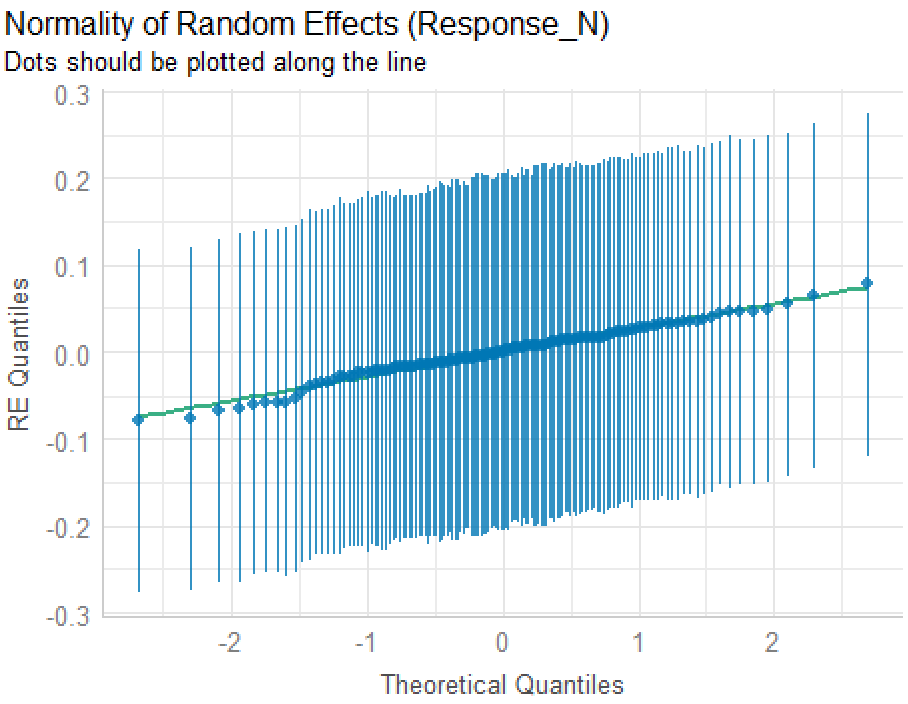
Normality of Random Effects (Response_N)
Dots should be plotted along the line
RE Quantiles
0.3
0.2
0.1
0.0
-0.1
-0.2
-0.3
-2
-1
0
1
2
Theoretical Quantiles

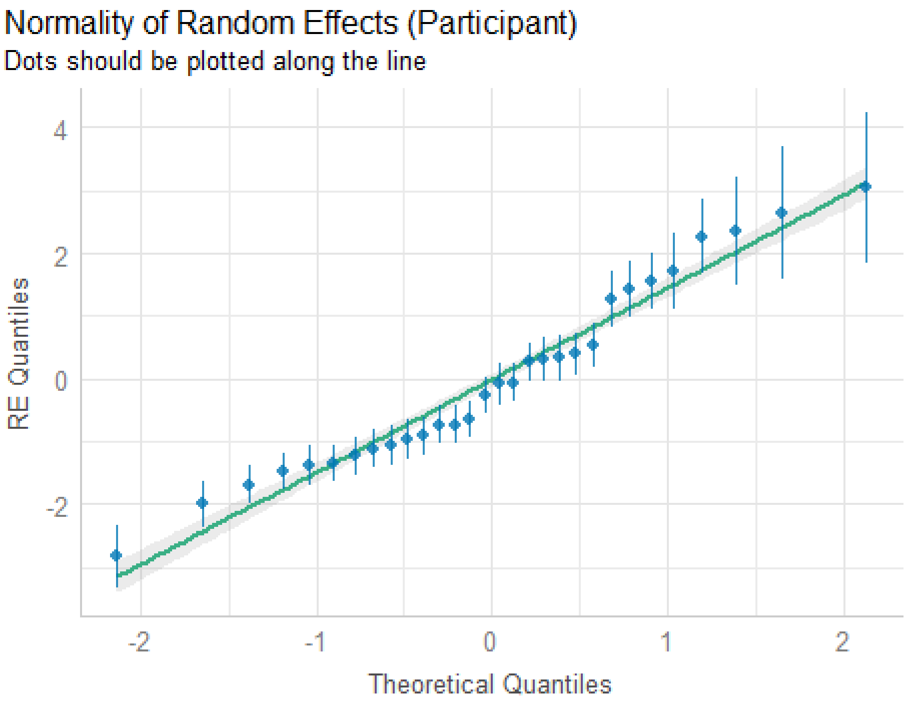
Normality of Random Effects (Participant)
Dots should be plotted along the line
RE Quantiles
4
2
0
-2
-2
-1
0
1
2
Theoretical Quantiles

Objective 3

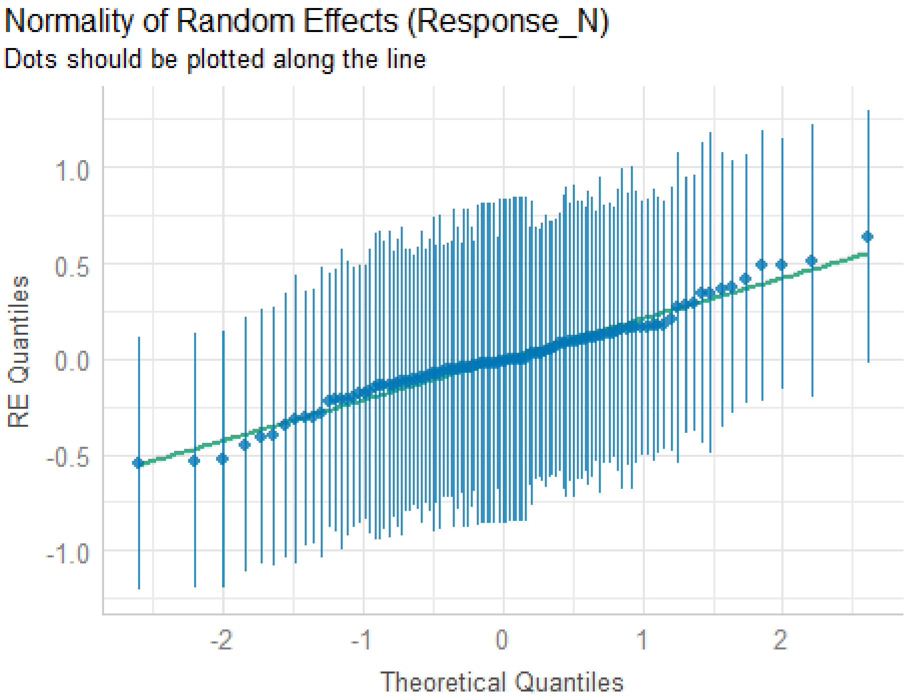
Normality of Random Effects (Response_N)
Dots should be plotted along the line
RE Quantiles
1.0
0.5
0.0
-0.5
-1.0
-2
-1
0
1
2
Theoretical Quantiles

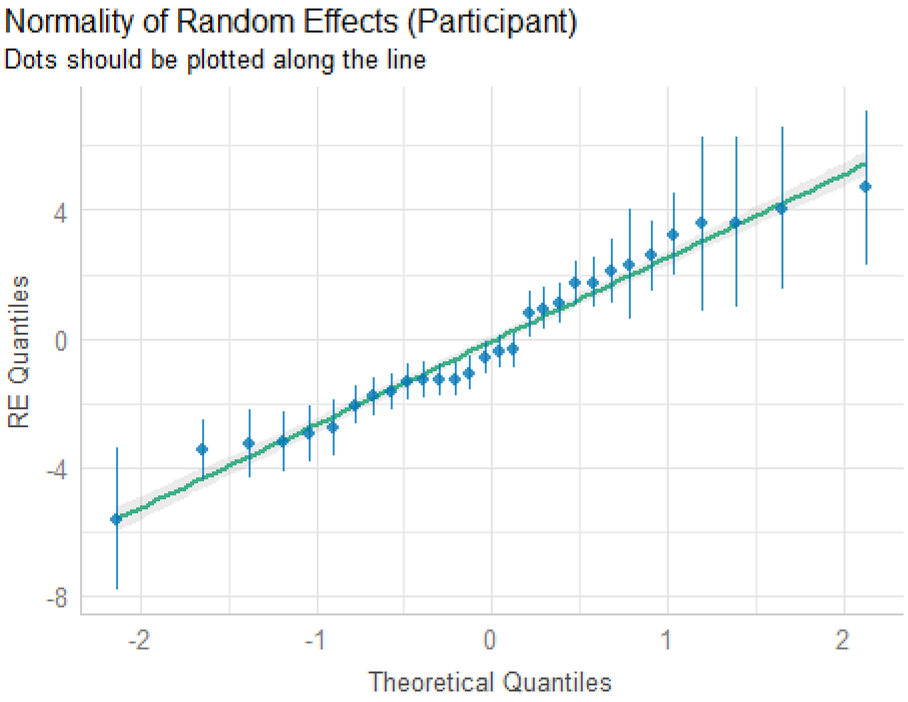
Normality of Random Effects (Participant)
Dots should be plotted along the line
RE Quantiles
4
0
-4
-8
-2
-1
0
1
2
Theoretical Quantiles
