# Supplementary material for: Sustainable behavior in the fishing cards digital game: a comparative analysis across extraction patterns
Source: Front Psychol. 2025 Apr 9;16:1507569. doi: 10.3389/fpsyg.2025.1507569 (PMC12015162; doi:10.3389/fpsyg.2025.1507569)
Supplement: Supplementary file 2 [file Presentation_1.zip › Supplementary material presentation/supplementary material A.docx]

**A - Instructions Presented During the Tutorial 1 (Translated from Brazilian Portuguese)**

1. "Welcome to the game 'Fishing Cards'. You are deep under the sea and will have the fun mission of catching fish using cards!"
2. "This tutorial will teach you how to play."
3. "Therefore, don't forget to read all the instructions the game will show."
4. "Click the button to start."
5. "Look at these two whirlpools."
6. "Fish will come out from the left side and try to reach the right side."
7. "Don’t let them enter the right whirlpool or they’ll escape!"
8. "Look! Fish are coming out of the whirlpool! Let’s learn how to catch them, okay?"
9. "Click on the panel with the mouse to generate a card."
10. "Press and hold the mouse button over the card, drag it, and take it to a fish."
11. "You used the Fishing Rod card. It only takes away part of the fish's life!"
12. "You need to use more of them to catch a fish."
13. "Keep trying to catch a fish."
14. "Great! Now on the panel, we have the Radar card, let’s use it!"
15. "You used the Radar card."
16. "It’s very strong and catches a fish instantly!"
17. "Cool! You've already used both types of cards."
18. "Now you can choose which one to use to catch the remaining fish."
19. "Good job! You caught all the fish! That's it!"
20. "Keep an eye on the panel with THREE HEARTS."
21. "They indicate your Life Points, and every time a fish escapes through the whirlpool, you lose a heart."
22. "If you lose three hearts, you lose the game!"
23. "Keep an eye on this clock. It indicates the time you have to fish during the matches."
24. "When the time runs out, you will move on to the next match."
25. "Also, watch your points that will appear in the basket when you catch a fish."
26. "Now you’re going to play, don’t worry if you lose, it’s just practice!"
