# Supplementary material for: Sustainable behavior in the fishing cards digital game: a comparative analysis across extraction patterns
Source: Front Psychol. 2025 Apr 9;16:1507569. doi: 10.3389/fpsyg.2025.1507569 (PMC12015162; doi:10.3389/fpsyg.2025.1507569)
Supplement: Supplementary file 2 [file Presentation_1.zip › Supplementary material presentation/supplementary material B.docx]

**B - Instructions Presented to the Player During Tutorial 2 (Translated from Brazilian Portuguese)**

1. "Now you will have two new challenges."
2. "Therefore, don't forget to read all the instructions the game will show."
3. "Click the button to start."
4. "Keep an eye on this green bar. It indicates the fish you have to catch."
5. "It decreases as you catch fish, but from time to time, the bar increases as new fish are born."
6. "If the bar runs out, you also lose the game!"
7. "New feature! The ocean fish will be shared with other players."
8. "They control submarines and will occasionally catch the same fish as you."
9. "Since the fish belong to everyone, if they catch them, the green bar will also decrease!"
10. "Now you are aware of the new challenge."
11. "Stay focused on the green bar, and be careful not to let the fish escape!"
12. "Click the button to return to the selection screen and good luck in scenario B."
